# Relationship between traumatic events, somatization, psychological distress, and quality of life in female refugees in Germany

**DOI:** 10.3389/fpsyt.2025.1627665

**Published:** 2025-11-26

**Authors:** Jenny Jesuthasan, James K. Moran, Eva J. Döring-Brandl, Ingar Abels, Christine Kurmeyer, Annabelle Starck, Ulrich Stangier, Jana Gutermann, Ulrike Zier, Anja Wollny, Antje Krüger, Kneginja Richter, Sabine Oertelt-Prigione, Meryam Schouler-Ocak

**Affiliations:** 1 Department of Psychiatry and Psychotherapy, St. Hedwig Hospital, Charité-Universitätsmedizin Berlin, Berlin, Germany; 2 Department of Psychiatry and Psychotherapy, Multisensory Integration Lab, Charité Universitätsmedizin, St Hedwig Hospital, Berlin, Germany; 3 Office of the Equal Opportunities Officer, Charité–Universitätsmedizin, Berlin, Germany; 4 Clinical Psychology and Psychotherapy, Department of Psychology, Goethe-University Frankfurt, Frankfurt, Germany; 5 Institute of Occupational, Social, and Environmental Medicine, University Medical Center of the Johannes Gutenberg University, Mainz, Germany; 6 Institute of General Practice, University Medical Center Rostock, Rostock, Germany; 7 University Clinic for Psychiatry and Psychotherapy, Paracelsus Medical University Nuremberg, Nuremberg, Germany; 8 Faculty for Social Sciences, Technical University Nuremberg Georg Simon Ohm, Nuremberg, Germany; 9 CuraMed Tagesklinik Nürnberg GmbH, Nuremberg, Germany; 10 Institute of Legal Medicine, Charité-Universitätsmedizin, Berlin, Germany; 11 Department of Primary and Community Care, Radboud Institute for Health Sciences, Radboud University Medical Center, Nijmegen, Netherlands

**Keywords:** female refugees, traumatic events, psychological distress, somatization, quality of life

## Abstract

**Theoretical background:**

Female refugees often experience traumatic events leading to mental health problems such as depression, anxiety, and somatization which can impact their quality of life. However, the interplay between these factors has rarely been studied to date.

**Objectives:**

This study investigates factors that contribute to the symptoms of psychological distress, somatization, and quality of life and the relationship between them.

**Methods:**

594 female refugees in five different reception centers in Germany were included. The cumulative number of traumatic events experienced was defined using the Harvard Trauma Questionnaire and the Posttraumatic Diagnostic Scale. Information from the Hopkins Symptom Checklist, the Symptom Checklist-90, and Eurohis-QoL was used to perform hierarchical regression analysis and serial mediation analysis was conducted using PROCESS Model 6 and bootstrapping.

**Results:**

Traumatic experiences had an impact on quality of life, both independently and as well as being mediated by somatization and psychological distress. Furthermore, our study reveals that cumulative trauma influences somatization, which then mediates psychological distress, ultimately leading to a lower quality of life. Cumulative trauma had the highest negative impact on mental health outcomes.

**Conclusion:**

Our findings suggest that future research and treatment of female refugees should focus on the role of somatization. Clinical assessments should be broadened with culturally sensitive tools to encompass both somatic and psychological dimensions of distress.

## 1 Introduction

At the end of 2024, approximately 123.2 million people worldwide were forcibly displaced or stateless due to armed conflicts, violence, and human rights violations. Germany was the third largest recipient of new individual applications worldwide (1). The largest group consisted of Ukrainian nationals, followed by individuals from Syria, Afghanistan, Iraq and Turkey. These five nationalities accounted for nearly three-quarters of all protection seekers residing in Germany (2). Approximately 50% of the refugees worldwide are estimated to be girls and women (1); however, the majority of refugees arriving in Europe –with exemption of Ukrainians- are male (2). Two potential reasons for the gender gap are: high smuggling costs prevent families from fleeing together, and many choose to protect women and children from the dangerous journey. Also, women are not allowed to travel alone and families often sent one man ahead, hoping to reunite later through legal family reunification. Men leave the country to evade compulsory military conscription or as a consequence of having deserted military service (3, 4).

While these figures highlight especially the scale of female refugee displacement, there are gender specific implications. Besides common war and conflict related stressors like lack of shelter, food or hygiene facilities there is a wide range of traumatic events and trauma types that in particular, women frequently experience: Severe war-related trauma such as torture, imprisonment, and life risk (5) as well as sexual or gender-based violence (6). Twice as many women reported exposure to family violence and sexual abuse compared to men (7). Female refugees also face social repercussions, including rejection or isolation, discrimination (e.g. for wearing a veil), divorce, abuse, “honor” crimes, and ostracism (8). Without a partner, they often lack protection and social support (9–11). A person’s vulnerability to developing mental disorders is increased by exposure to traumatic events (10).

Traumatic experiences have profound implications for mental health outcomes. Refugees suffer more often from mental health disorders as compared to the general population. In post-conflict settings, more than one in five people show signs of mental health problems (12). In response to pre-, peri- and postmigration trauma and acculturative stress, they can develop a wide range of emotional, cognitive, physical, behavioral, and social problems and have high prevalence for mental disorders (8, 13–17). As we previously reported (18), cumulative exposure to potentially traumatic events strongly associated with depression (13, 19, 20), somatization (21) and Posttraumatic stress disorder (PTSD) (22). The risk to develop the latter is influenced by the type of trauma, for example interpersonal violence or self-experienced traumatic events (15, 23). However, prevalence estimates vary greatly (24), possibly due to methodological differences and refugee characteristics (25).

These mental health challenges are frequently expressed through somatic complaints highlighting the complex interplay between mind and body in refugee populations. Somatization describes the expression of physical symptoms that a medical examination cannot explain (26). While PTSD, depression, and anxiety are well studied, there is little research on somatic symptoms, although somatic stress disorder is also related to trauma exposure (26–28), emotional distress and problems in functioning. Somatization is very common among refugees and often drives their increased utilization of health services, to address apparently physical complaints (29). For example, Syrian participants reported various somatic symptoms, like cardiovascular and pulmonary symptoms, severe pain in different body parts, hair loss, or even unfulfilled desire to have children (30). Yazidi women who escaped from the Islamic State (IS) reported feelings of suffocation, movement disorders, back pain, headaches, internal pain, heavy movement, heavy legs, kidney and eye problems, hair loss and anger (31).

The challenge of distinguishing somatization from other mental illnesses highlights the complexity of mental health evaluations in refugee populations. Symptoms of PTSD, anxiety, depression, and somatic symptoms can cluster, making differentiation between the different entities difficult (29, 32, 33). The frequent co-occurrence of psychological distress and pain is driven by complex mechanisms that are likely bidirectional and influenced by both genetic and environmental factors (34). Somatization is often associated with trauma, but the causal direction of the correlation between PTSD and somatization is unclear and there is uncertainty about the presence of a potential third mediator variable. One hypothesis is that psychological stress caused by PTSD increases the vulnerability to develop somatic symptoms (35). Somatization might also be the preferred suffering expression mode in certain populations (36). Another hypothesis is that past traumatic bodily experiences are stored in memory and influence the development of somatic symptoms (37).

Traumatization may lead to mental health challenges including somatization, consecutively impacting the quality of life for refugees. Increased mental health disorders and somatic complaints could be a reason why refugees experience lower quality of life compared to international population norms. For example, high somatization of mental disorders or current physical pain due to past torture experiences might explain lower scores in the physical health domain of quality of life (38, 39). Furthermore, the relationship between trauma and quality of life was mediated by somatic complaints among Tunisians during the Arab Spring and among Ivorian Refugees (40).

Being a woman is a significant and common sociodemographic risk factor among refugees (29). Female refugees and asylum seekers report more frequent and severe somatic, depressive, and anxiety symptoms compared to males (33, 41, 42). Female refugees are a vulnerable group and Vallejo-Martín et al. (23) propose several reasons why addressing their mental health in studies must be done sensitively and with care: The risk of traumatization, concentration problems, language barriers, discrimination, social rejection, or addressing taboo topics like rape and sexual assaults (23). Research on women remains underreprensented. This underscores the importance of focusing research and interventions on female refugees, whose unique experiences and mental health needs often differ significantly from those of their male counterparts.

The relationships between trauma, mental health problems and quality of life in female refugees is not well understood. This study aims to explore the influence of sociodemographic variables and cumulative traumatic experiences on the severity of mental distress, somatization, and life quality. Furthermore, we examine the link between experienced traumatic events, somatization, psychological distress, and quality of life. Though similar relationships have been explored in the literature, this research seeks to shed new light on the complex interplay between these variables, offering an original angle by examining sequential mediation effects. We hypothesized that traumatic experiences, somatic symptoms, and psychological distress would be associated with a lower quality of life. We also predict that somatization and psychological distress have mediating roles in the relationship between trauma and quality of life.

## 2 Methods

### 2.1 Procedure

Data was obtained between August and December 2016 during a 12-month multicenter study, the “Female Refugee Study”, published elsewhere (43). Briefly, female participants were recruited according to the quota for the country of origin based on statistical distribution provided by the Federal Office for Migration and Refugees (BAMF). The participants were over 18 years of age and their countries of origin were Afghanistan, Syria, Iran, Iraq, Somalia, and Eritrea. These nationalities were selected based on the likelihood of granting them refugee status at time of recruitment. They lived in shared reception facilities and were recruited by native speakers at informal events or through direct informal invitations in five German regions (Berlin, Bavaria, Mecklenburg-Western Pomerania, Hesse, and Rhineland-Palatinate). The sample size was estimated using an *a priori* power analysis to achieve a statistical power of 1–β = .80. A total of 663 women were recruited. We excluded cases with missing answers as described in the data analysis section. Written informed consent in English, Arabic, Farsi, Somalia, and Tigrinya was obtained in advance to the study. These languages were an inclusion criterion for participation based on the selected nationalities. Interviews with participants were conducted at least 24 after they had provided their consent. The interviews were one-on-one interviews, which lasted up to two and a half hours. Literate participants completed the questionnaires themselves, supported by the interviewer for open questions; for the others, the interviewer read the questions and provided written responses on their behalf. The interviewers were native speakers (Arabic, Dari/Farsi, Somali and Tigrinya) and were trained to conduct the survey with traumatised individuals and to use standardised instructions and information to guide participants through the questionnaire. They also participated in team meetings and supervision by telephone or in person throughout the study. The questionnaire was translated into Arabic, Farsi, Somali, and Tigrinya by a native speaker and back translated by a different native speaker, then compared with the original and discussed with the interpreters before implementation. All project partners obtained ethical approval (reference numbers are Berlin: EA1/117/16, Nuremberg: 016/1511, Rostock: A2016-0142, Frankfurt a. M.: 334/16, Mainz: 837.316.16 (10635)). All procedures were in accordance with the declaration of Helsinki. Participants were only asked one follow-up question if they did not answer a question. They could skip the question if they did not want to respond. This led to missing data. In the following analysis, women with more than one missing answer in the traumatic experience items were excluded.

### 2.2 Measures

#### 2.2.1 Psychological distress - HSCL

The Hopkins Symptom Checklist (HSCL) has a long history back to the 1950s (44) and several checklists (21–90 items) derived from it. The HSCL-25 employs the scales of anxiety and depression from the HSCL-58. These dimensions are a sensitive measure for global psychological distress. The symptom checklist assesses anxiety with 10 items and depression with 15 items. Response options for items ranged from 1 to 4. The total score refers to the resulting global psychological distress (45). If there are more than 10% of missing answers per scale, the test should not be evaluated (46). It has been used in cross-cultural settings investigating the mental health of immigrants, refugees, or asylum seekers and was translated into several languages. The internal consistency for the total score has shown a high Cronbach’s Alpha of.94 in previous research (47). In the current study, Cronbach’s alpha was.93.

#### 2.2.2 Somatization - SCL SOMA

The SOMA subscale of the Symptom-Checklist-90 assesses symptoms of somatization including physical stress and functional disorders using 12 items on a 5-point Likert scale. The maximum value of missing items is 4 items (48). The reliability is high: Cronbach’s alpha was.83. in prior studies (49). Cronbach’s alpha in our study was.89.

#### 2.2.3 Number of traumatic events - HTQ/PDS

For assessment of the exposure to traumatic events, we used part of the Harvard Trauma Questionnaire (HTQ) (50), which is a cross-cultural screening instrument to document trauma exposure and trauma-related symptoms in refugees, which also includes culturally based expressions of distress (51, 52). This instrument was developed specifically in a refugee sample and has shown excellent statistical properties (Cronbach α = .90) (53). The HTQ has often been modified in studies and items have been added, amended, or removed (54). The earliest version of the HTQ contained four answer options (E = experienced, W = witnessed, H = heard about, or N = no), in the revised version of the HTQ response options were reduced to “Yes” or “No” and the “witnessed” option was only retained where relevant. Based on the revised version, we recoded the items and reduced the answer options to “experienced” and “witnessed” (for the items: “attacks on family members and strangers” and “torture”). Mollica recommends establishing possible torture and trauma events in different cultural groups and geopolitical situations (55). In later versions, Part I was expanded and included 46 to 82 traumatic events (56). We combined traumatic events with events from the Posttraumatic Diagnostic Scale (PDS), a self-report measure of PTSD with a high internal consistency with Cronbach’s alpha.92 (57), which was also already used in an Arabic version for the immigrant and refugee population (Cronbach’s alpha.93) (58). Cronbach’s alpha in our study was.80.

#### 2.2.4 Quality of life – eurohis-QoL

The Eurohis-QOL index is an adaptation of World Health Organization Quality of Life questionnaire (WHOQOL-100) and the World Health Organization Quality of Life Brief Version (WHOQOLBREF) and is a self-assessment instrument of generic quality of life (59). An overall score is formed with 8 items for the domains psychological, physical, social, and environmental well-being which are each represented by two items. Answer options range from “not at all” to “completely” on a 5-point response format on a Likert scale (60). The instrument showed acceptable cross-cultural performance with internal consistency measured using Cronbach’s alpha ranging between.72 and.81 across countries (61) and was also used with refugee populations (62). For this analysis, we had a Cronbach Alpha of.78.

#### 2.2.5 Data analysis

We conducted hierarchical multiple regression analysis to examine the stepwise overall association between variables. More specifically, we conducted three hierarchical linear regression analyses, initially examining sociodemographic variables’ predictive power and subsequently adding the number of traumatic events to predict somatization, psychological distress, and quality of life outcomes. The relevant assumptions of this statistical analysis (linearity, no outliers, independence of residuals, no multicollinearity, homoscedasticity, normally distribution) were checked and fulfilled the required criteria. The variables were chosen based on previous research, suggesting sociodemographic variables such as age, cohabitation (living with a husband or partner), mothers with children (mothers who are living together with their children), mothers without children (mothers who are not living together with their children), secondary education (visited school or having vocational training), higher education (studied or having a doctorate degree), language proficiency in English/German skills (knowledge of German and/or English language), and cumulative traumatic events are important determinants of somatization, psychological distress, and quality of life (5, 63–66). The variables did not follow any order. We used Serial Multiple Mediation Model 6 for the serial mediation model with the PROCESS macro plug-in application by Hayes (67). This is based on a bootstrapping approach with 10,000 bootstrap samples and was used to test the mediation effects of psychological distress and somatization in the link between traumatic experiences and quality of life. Hayes (67) states that the bootstrapping method is statistically more powerful than other mediation techniques (e.g., Sobel test). Ordinary least squares (OLS) analysis was used to estimate unstandardized path coefficients for total, direct, and indirect effects (67). We adjusted for the effect of the sociodemographic correlate age.

We used a statistical significance of 0.05 (two-tailed p-value) and a 95% percentile bootstrap confidence interval (PBCI). Given the exploratory nature of our study, we used a two-tailed test to allow for the detection of effects in both directions, acknowledging that a one-tailed test could fail to identify existing effects if the true direction of the relationship differs from the predicted one (68). Mediation is significant if the lower limit and upper limit for the indirect effect do not include zero (69, 70). Assuming bidirectional associations, both possible mediation sequences have been explored: psychological distress influencing somatization and vice versa. We excluded cases with more than one missing answer on the anxiety scale and more than two missing answers on the depression scale of the HSCL-25. For SCL-SOMA we set a cut-value of 20% for missing answers and for EUROHIS-QoL we did a listwise deletion. We used the SPSS Software package (Version 26, IBM Corp., Armonk, NY, USA) and PROCESS macro for SPSS v3.5.

## 3 Results

### 3.1 Sociodemographic characteristics and measure statistics

We included 594 women in the final analyses. Most came from Syria (49.0%, n=291) and Afghanistan (24.9%, n= 148). The participant’s mean age was 32.38 years (SD = 10.28). 80% (n=475) of the women stated they have children. 65.3% (n=388) of the women lived together with their husband or partner. 66.3% had attended school or had vocational training, 17.2% started or finished their studies or had a doctorate. 217 women spoke English, German, or both languages (see **Table 1**).

**Table 1:** Characteristics of the sample.

Characteristics	*M*	*SD*	Range	*n*	%
Country					
Afghanistan				148	24.9
Eritrea				394	6.6
Iraq				70	11.8
Iran				27	4.5
Somalia				19	3.2
Syria				291	49
Age	32.38	10.28	17-69	592	99.7
Cohabitation					
Living without partner or husband				206	34.7
Living with a partner or husband				388	65.3
Education^a^					
No education				96	16.2
Secondary education				394	66.3
Higher education				102	17.2
Parenthood^a^					
No children				114	19.2
Mothers living with children				420	70.7
Mothers living without children				55	9.3
Number of children	2.26	1.88	0-10		
Language skills					
No German-English				377	63.5
German-English				217	36.5
Number of traumatic events	5.69	4.16	0-24		
SCL	2.2	0.92	1-5		
EHQ	3.22	0.77	1-5		
HSCL	2.25	0.71	1-3.82		

N = 594. ^a^Reflects the number and percentage of participants answering this questions.

SCL = Somatization; EHQ = Quality of Life; HSCL = Psychosocial Distress.

### 3.2 Predicting symptoms of somatization, psychological distress and quality of life

Separate hierarchical multiple regression analysis was performed to examine the predictive value of demographic variables and the number of traumatic events in somatization, psychological distress, and quality of life (**Table 2**). In step 1, we entered the predictor variables age, cohabitation, secondary education, higher education, mothers with children, mothers without children, and German/English skills. These variables explained 5% of the variance of somatization, *F*(7, 577) = 4.60, *p* <.001. In step 2, the number of traumatic events (NTE) was a significant positive predictor and explained an additional 11% for somatization, *F*(1, 576) = 72.76, *B* = 0.08, β = .34, p <.001, Δ *R^2^* = .11. Age remained significant in both steps.

**Table 2:** Summary of hierarchical regression analysis for variables predicting somatization, psychological distress and quality of life.

	Variable	Somatization				Psychological distress				Quality of life			
		*B*	*SE B*	β	*p*	*B*	*SE B*	β	*p*	*B*	*SE B*	β	*p*
Step 1	Constant	1.83	0.18		<.001***	2.03	0.14		<.001***	3.55	.16		<.001***
	Age	0.02	0.00	.18	<.001***	0.01	0.00	.08	.08	-0.01	0.00	-.08	.07
	Cohabitation ^a^	-0.09	0.09	-.05	.29	-0.18	0.07	-.12	.011**	0.16	0.08	.10	.036*
	Secondary education ^b^	-0.20	0.11	-.10	.06	0.04	0.09	.03	.66	-0.14	0.10	-.09	.13
	Higher education ^c^	-0.27	0.14	-.11	.06	-0.03	0.11	-.02	.80	-0.26	0.13	-.13	.039*
	Mothers with children ^d^	0.07	0.11	.04	.51	0.12	0.09	.08	.16	-0.13	0.10	-.08	.19
	Mothers without children ^e^	0.14	0.16	.04	.39	0.15	0.13	.06	.25	0.00	0.14	.00	.98
	German-English ^f^	0.11	0.09	.06	.20	0.08	0.07	.05	.27	0.03	0.08	.02	.68
Step 2	Constant	1.388	0.18		<.001***	1.685	0.14		<.001***	3.738	0.16		<.001***
	Age	0.02	0.00	.17	<.001***	0.01	0.00	.07	.10	-0.01	0.00	-.08	.09
	Cohabitation ^a^	0.01	0.08	.01	.87	-0.09	0.07	-.06	.18	0.12	0.08	.07	.13
	Secondary education ^b^	-0.13	0.10	-.07	.19	0.09	0.08	.06	.25	-0.17	0.09	-.11	.07
	Higher education ^c^	-0.13	0.14	-.06	.32	0.07	0.11	.04	.50	-0.31	0.12	-.15	.012*
	Mothers with children ^d^	-0.02	0.11	-.01	.87	0.05	0.08	.03	.59	-0.08	0.10	-.05	.40
	Mothers without children ^e^	0.02	0.15	.01	.90	0.05	0.12	.02	.71	0.06	0.14	.02	.65
	German-English ^f^	0.06	0.08	.03	.47	0.03	0.06	.02	.59	0.05	0.08	.03	.47
	NTE	0.08	0.01	.34	<.001***	0.06	0.01	.35	<.001***	-0.04	0.01	-.18	<.001***
	*R* ^2^	05***				03*				.03*			
	Δ *R*^2^	.11***				.11***				.03***			
	F for Δ*R*^2^	13.61				11.65				4.28			

*N* = 594. *p <.05; **p <.01; ***p < 0.001.

^a^Living without partner or husband vs. living with a partner or husband. ^b^No education vs. visited school/vocational training. ^c^No education vs. studied or having a doctorate degree. ^d^No children vs. mothers living with child/children. ^e^No children vs. mothers not living with their child/children. ^f^No German or English skills vs. German or English skills.Bold values indicate statistically significant results.

Repeating the same procedure for the dependent variable psychological distress, the model explained 3% of the variance, *F* (7, 560) = 2.43, *p* = .019, specifically that living with a partner decreased the score for distress (*B = - 0.18*, β *= -.12, p* = .011). In step 2, the number of traumatic events was the only significant positive predictor and explained an additional 11% for psychological distress, *F*(1, 559) = 73,98, *B* = *0.06*, β *= .35*, p <.001, Δ *R^2^* = .11.

Examining quality of life as a dependent variable in the third model, in the first step, 3% of the variance was explained, *F*(7, 552) = 2.15, *p* = .037. Having a partner (*B = 0.16, β = .10, p* = .036) and having higher education (*B = -0.26, β = -.13, p* = .039) were significant predictors, suggesting that having a partner predicted increased quality of life and having a higher education predicted lower quality of life. In step 2, the number of traumatic events was a significant negative predictor for quality of life and explained an additional 3% for quality of life *F*(1, 551) = 18.65, {it}B = -0.04,

β = -.18, {/it}p <.001, Δ *R^2^* = .03. Even though living together with a partner or husband predicted significant decreases in psychological distress and increased quality of life in step 1, it had no significant influence on the fully adjusted model. However, having a higher education remained a significant predictor, which reduced perceived quality of life. For all regressions, the number of traumatic events was the strongest predictor.

### 3.3 Mediating effects of somatization and psychological distress in the relationship between trauma and quality of life

**Figure 1:**
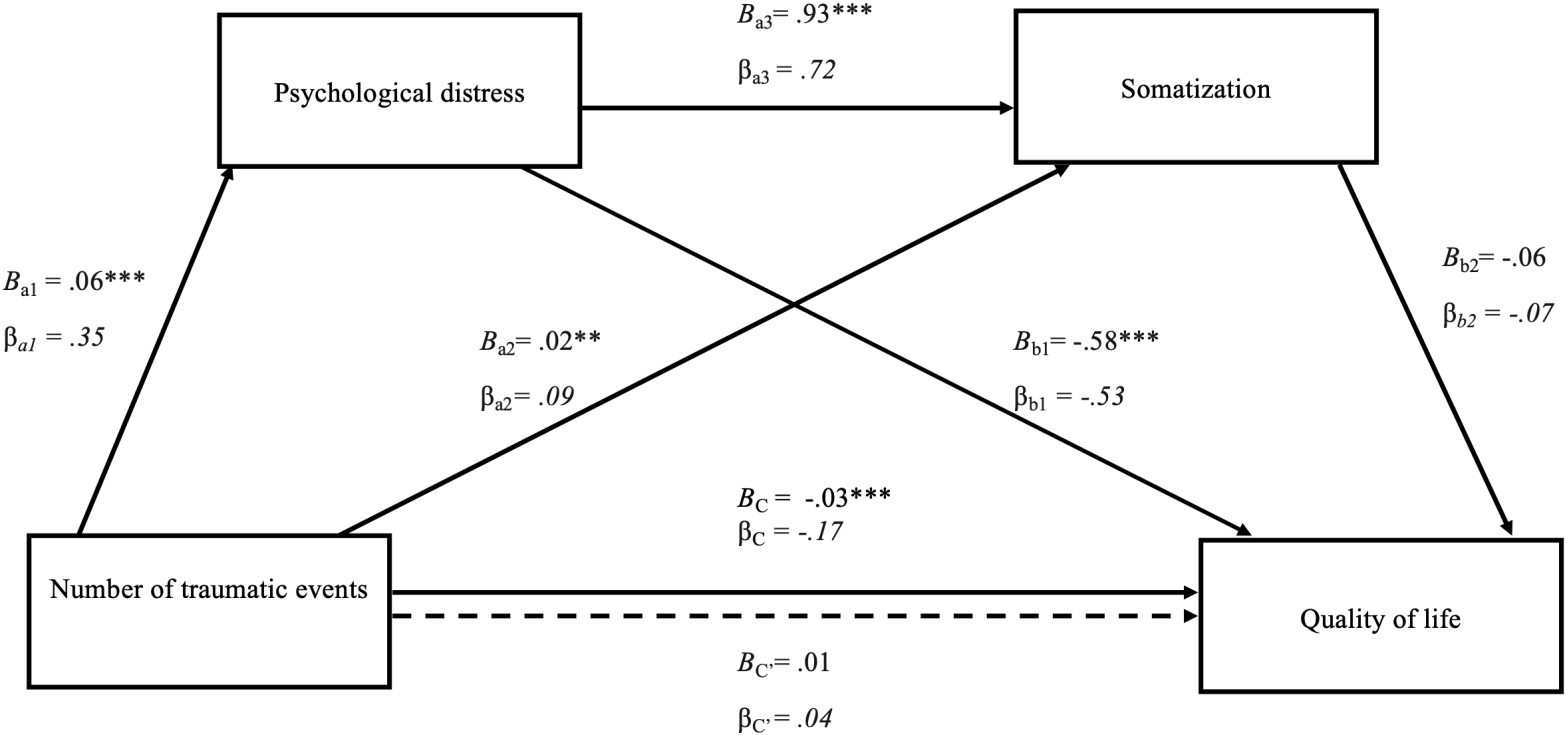
Serial mediation model of the effect of number of traumatic events on quality of life through psychological distress and somatization. The figure displays the serial multiple mediation of psychological distress and somatization in the relationship between the number of traumatic events and quality of life. Path coefficients are presented as unstandardized (B) and standardized (β) values. The total effect (C) and direct effect (C′) of the number of traumatic events and quality of lif are also shown. *p <.05, **p < 0.01, ***p < 0.001.

**Figure 2:**
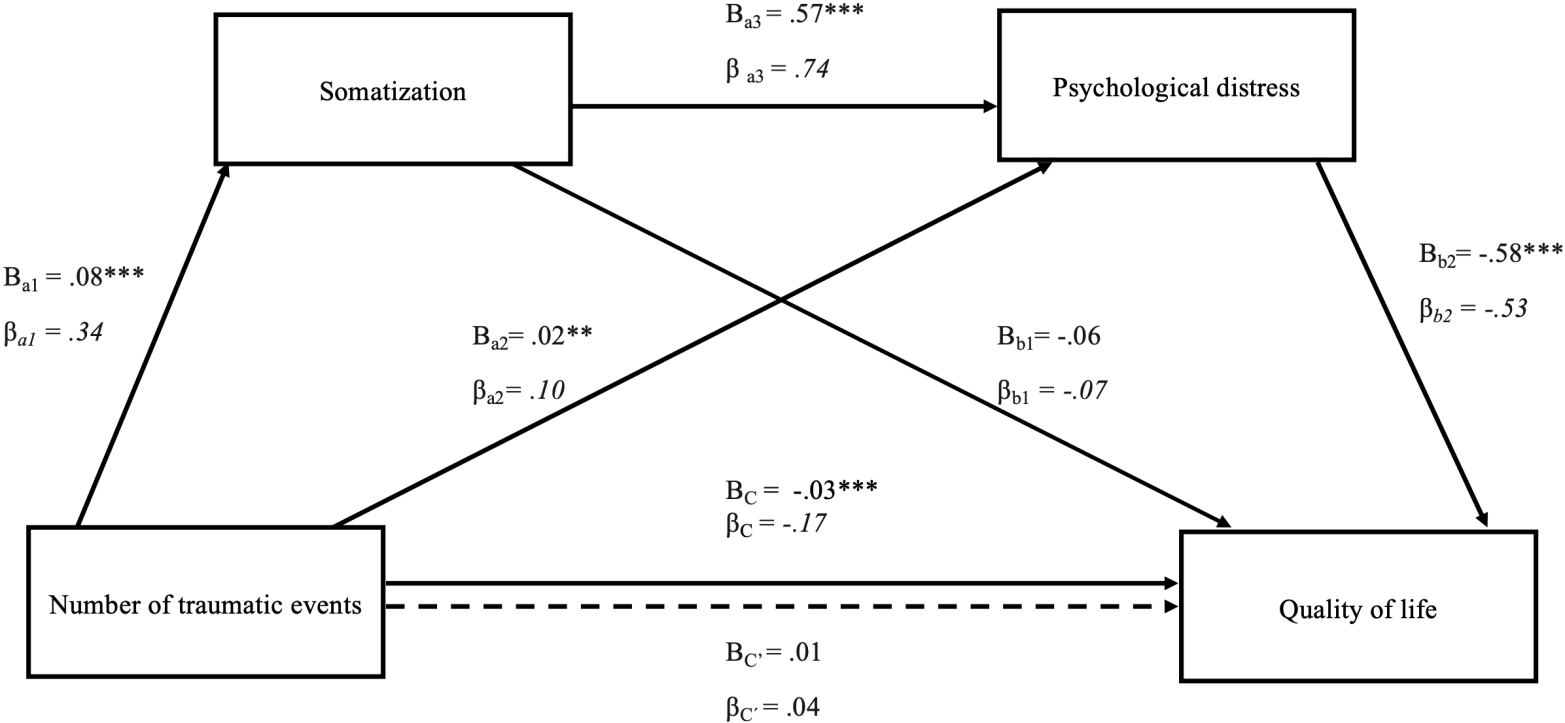
Serial mediation model of the effect of number of traumatic events on quality of life through somatization and psychological distress. The figure displays the serial multiple mediation of somatization and psychological distress in the relationship between the number of traumatic events and quality of life. Path coefficients are presented as unstandardized (B) and standardized (β) values. The total effect (C) and direct effect (C′) of the number of traumatic events and quality of life are also shown. *p <.05, **p < 0.01, ***p < 0.001.

**Figures 1**, **2** present the findings of the two tested models of the mediating roles of somatization and psychological distress in the relationship between the number of traumatic events and quality of life. The total effect, *C* = -0.03 *SE* = .01, *t*(550) = -3.82, β = -.17, *p* <.001, of traumatic events on quality of life was significant, indicating that people with a higher number of traumatic experiences show lowered perception of quality of life.

The first analysis with psychological distress and somatization as mediators is presented in **Figure 1**. The number of traumatic events was associated with psychological distress, *B* = 0.06 *SE* = .01, t(550) = 8.03, *p* < 0.001, β = .35, suggesting that psychological distress increased with the number of traumatic events. Furthermore, psychological distress had a significant direct effect on quality of life, *B* = -0.58, *SE* = 0.07, *t*(548) = -8.65, β = -.53, *p* <.001, as well as maintaining a significant indirect effect (a_1_ and b_1_): *B* = -0.04, 95% CI [-0.047, -0.024], β = -.18. Although both the direct effects of traumatic events, *B* = 0.02, *SE* = .001, *t*(549) = 3.03, β *= .*09*, p* = 0.003, and psychological distress were associated with somatization, *B* = 0.93, *SE* = .04, *t*(549) = 22.66, β *= .*72*, p* <.001, the direct effect of somatization on quality of life was not significant *B* = -0.06, *SE* = .05, *t*(548) = -1.11, β *=* -.07*, p* = 0.27, resolving in no indirect effect (a2 and b2): *B* = -0.00, 95% CI [-0.004, 0.001], β = .01 or serial mediation effect (a1a3b2): *B* = -0.00, 95% CI [-.009,.002], β = -.02.

These results indicate that psychological distress, the sole mediator, was mediating between the number of traumatic events and quality of life.

The same analysis replacing psychological distress with somatization (see **Figure 2**) showed that the direct effect of traumatic experiences on somatization, *B* = 0.08, *SE* = .01, *t*(550) = 8.19, β = .34, *p* < 0.001 was significant. This suggests that somatization increased with the number of traumatic events. Somatization as a first mediator was associated with higher psychological distress, *B* = 0.57, *SE* = .02, *t*(549) = 27.19, β = .74, *p* < 0.001, which finally acted as a second mediator *B* = -.58, *SE* = .07, *t*(548) = -8.65, β = .53, *p* <.001, resolving in a serial mediation between the number of traumatic events and quality of life, indirect effect (a1 a3 b2): *B* = -0.03 95% CI [-.034, -.017], β = -.13.

Similarly, the number of traumatic events were associated with psychological distress, *B* = 0.02, *SE* = .01, *t*(549) = 3.11, β = .10, *p* < 0.002, which acted as mediator, *B* = -0.58, *SE* = .07, *t*(548) = -8.65, β = -.53, *p* <.001, between the number of traumatic events and quality of life, indirect effect (a2 and b2): *B* = -0.01, 95% CI [-.017, -.004], β = -.05.

The statistical significance or non-significance of indirect effects are presented in **Tables 3**, **4**. They present the specific indirect effects of the number of traumatic events through somatization and psychological distress on quality of life. To compare the specific indirect effects of the two significant paths, NTE-SOM-PD-QoL and NTE-PD-QoL from model 2, the specific indirect effect contrast is presented in **Table 4**. This contrast shows if the two paths differ significantly based on a percentile-corrected confidence interval of 95%. Since zero is not contained in the interval, the two indirect effects can be distinguished. The mediating role of psychological distress alone was weaker than the serial multiple mediation role of somatization and psychological distress together.

**Table 3:** Specific indirect effects of number of traumatic events through psychological distress and somatization on quality of life.

Effect	Products of coefficients		Percentile bootstrap 95% CI^1^		
	*B*	*SE*	Lower limit	Upper limit	β
Total Indirect Effect	-0.039	0.005	-0.049	-0.029	-.21
NTE → PD →QoL	-0.035	0.006	-0.047	-0.024	-.18
NTE → SOM →QoL	-0.001	0.001	-0.004	0.001	-.01
NTE → PD → SOM →QoL	-0.003	0.003	-0.009	0.002	-.02

Number of bootstrap samples for percentile bootstrap intervalls: 10,000.

NTE = Number of traumatic events. PD = Psychological Distress. SOM = Somatization. QoL = Quality of Life.

^1^Confidence Interval.

To retain interpretability of small effect directions, values are reported to three decimal places; rounding to two would have led to values of +.00 or –.00.

**Table 4:** Specific indirect effects of number of traumatic events on quality of life.

Effect	Products of coefficients		Percentile bootstrap 95% CI^1^		
	*B*	*SE*	Lower limit	Upper limit	β
Total Indirect Effect	-0.039	0.005	-0.049	-0.029	-.05
NTE → SOM →QoL	-0.004	0.004	-0.012	0.003	-.01
NTE → PD →QoL	-0.010	0.003	-0.017	-0.004	-.01
NTE → SOM → PD →QoL	-0.025	0.004	-0.034	-0.017	-.03
Path1-Path3	0.021	0.007	0.008	0.035	.11
Path2-Path3	0.015	0.005	0.007	0.025	.08

Number of bootstrap samples for percentile bootstrap intervalls:10,000.

NTE = Number of traumatic events. PD = Psychological Distress. SOM = Somatization. QoL = Quality of Life.

^1^Confidence Interval.

To retain interpretability of small effect directions, values are reported to three decimal places; rounding to two would have led to values of +.00 or –.00.

The pathways between somatization and psychological distress are bidirectional. In the first model, psychological distress is correlated with somatization, *B* = 0.93, *SE* = .04, *t*(549) = 22.66, β *= .*72*, p* <.001, suggesting that more psychological distress leads to more somatization. In the second model, somatization is correlated with psychological distress, *B* = 0.57, *SE* = .02, *t*(549) = 27.19, β = .74, *p* < 0.001), suggesting that the more somatization symptoms are present, the higher the psychological distress.

## 4 Discussion

This study investigates the relationships between traumatic events, somatization, psychological distress, and quality of life of female refugees residing in Germany. We demonstrated that somatization and psychological distress have an influence on the relationship between trauma and quality of life. In this context, it’s important to highlight the observation established through serial mediation suggesting that cumulative trauma may impact quality of life sequentially by first influencing somatization, which then appears to influence psychological distress. A further result is that cumulative trauma emerges as the key predictor of increased somatization, psychological distress and reduced quality of life.

The finding that somatization and psychological distress independently mediated the relationship between trauma and quality of life, aligns with our initial research question and supports existing results by other researchers (36, 40, 71, 72), showing that a higher number of traumatic events is associated with somatization as well as psychological distress and further leads to lower quality of life. But beyond that, our study reveals that cumulative trauma affects somatization, which in turn influences psychological distress and finally contributed to a lower quality of life. This result suggests that it is important for clinicians to take into account the centrality of somatization for mental health, particularly since somatic symptoms often manifests as the initial presentation to clinicians. (29). Healthcare providers need to expand their understanding of the co-occurrence and potential interplay between psychological and somatic symptoms, especially when treating traumatized refugee patients. This may help to reduce misdiagnoses or failed treatment attempts due to unclear somatic symptoms. Clinical assessments should be broadened to incorporate integrated screening for both somatic and psychological distress (34).

The number of medically unexplained physical symptoms among refugees from non-Western countries is generally higher than among the general population (26). It is well-documented that PTSD coexists with somatic symptoms within the population of refugees. The association between PTSD and somatization is complex and not fully understood. It is possible that injuries suffered in traumatic events, such as torture or violence, explains this relationship, at least in part (73). Our previous study found that in addition to the overall level of trauma exposure, family violence experienced by refugee women played the most significant role in contributing to various symptoms, highlighting the critical impact of interpersonal trauma and insecurity (74). Within the current study, we did not differentiate between types of trauma and therefore can´t assess specific effects of family violence and other traumatic events.

Pain and distress are expressed in culturally specific ways, influenced by local beliefs, stigma, and accepted norms of suffering. In Syrian idioms of distress, for instance, the body and soul are seen as deeply interconnected, with physical symptoms often serving as valid expressions of emotional or psychological pain (34). Similarly, Hinton and Bui (75) show that experiencing of trauma differs radically across cultural groups, shaped by both universal and culturally specific dimensions of psychopathology. Their cross-cultural model, based on Cambodian populations emphasizes the importance of somatic symptoms, cultural syndromes, catastrophic cognitions, and culturally patterned worry and implies that trauma experience and recovery varies locally (75). Refugees may explain symptoms in ways that western professionals interpret as somatization (76) or nonspecific symptoms of PTSD. And, similar to torture survivors, pain from violence may be under-assessed (77).Our sample did not undergo a medical physical assessment. It is possible that participants in our sample still suffered from physical pain due to injuries experienced in their country or in flight, which was evaluated as somatic pain in our study.

This finding has implications for the diagnosis and treatment of refugees. Diagnosing and treating this population with the necessary sensitivity requires a deep and comprehensive understanding of women’s experiences of war and flight, as well as knowledge of partner and family violence. For the health assessment, the support of interpreters and written evaluation instruments in the native language is necessary. In the case of acute and chronic pain, a physician should give a clear assessment. The multifaceted aspects of chronic pain, pain physiology, mechanisms for chronification, and biopsychological model should be understood by the clinicians. Furthermore, the consequences of trauma and violence on mental health should be addressed and communicated in an understandable way to female refugees. This means that treatment options should be provided, and pain rehabilitation should be an option (76).

A recent meta-analysis by Sambucini et al. (78) on 52 studies spanning 24 years assessed the effectiveness of psychological treatments, for depression anxiety and somatization in migrants, including refugees. Cognitive-behavioral therapy emerged as the most effective. Notably, the number of studies addressing somatization were limited and underscore the necessity for additional research to evaluate the efficacy of psychological treatments for somatization in refugees (78). This is supported by a systematic review and network meta-analysis of 103 studies on psychosocial interventions for migrants which reported promising effects on PTSD, depression, and anxiety. However, due to generally low confidence in the evidence, no clear conclusions could be drawn (79).

We postulate that there is a bidirectional relationship between somatization and depression. In our mediation models, increased psychological distress is associated with increased risk for somatization and vice versa. This is in accordance with research showing that somatization and depression are often comorbid, but the causal direction is not clear (26, 29, 32–34).

As we found that cumulative trauma affected somatization, which in turn influenced psychological distress and finally contributed to a lower quality of life. Interestingly, the reverse prediction that cumulative trauma would influence quality of life via psychological distress and somatization was not supported. This sequence, where somatization comes before psychological distress, appears most compelling and may reflect a key pathway for early detection and intervention, with important implications for both research and clinical practice. A reason for this might be that somatic symptom disorder is characterized by enduring physical symptoms, which are not always attributed to a recognized medical condition (73). The inability to attribute pain to a physical cause and thus remain unexplained could trigger excessive thoughts, anxiety, and/or behaviors that lead to considerable distress.

We further found that the number of traumatic events was the most important predictor across all dependent variables. Also, the result that cumulative trauma is a predictor of somatization is consistent with previous findings (21). We confirmed that an increase in the number of traumatic events led to a higher overall psychological distress (10, 14) as we already reported in a previous study (18), which supports the dose-effect relationship hypothesis of cumulative trauma on psychiatric symptoms (9, 80). The quality of life decreased with an increasing number of traumatic experiences, which is consistent with a previous study (72) and has been confirmed in the serial mediation analysis.

Counterintuively, lower quality of life was also linked to higher education. Atrooz et al. (81) identified higher education as a key protective factor against psychological distress among Syrian female refugees, suggesting that it could serve as a buffer against mental health challenges (81). One possible explanation for our contrasting result is that highly educated female refugees face distinct disadvantages and systemic barriers in the labor markets such as job downgrading and economic penalties (82). In addition, they are more vulnerable to negative effects of discriminatory social attitudes, which can further diminish their overall quality of life (83).

The results of our study need to be interpreted with consideration of several limitations. First, we used cross-sectional data. The participants in our study represent women living in German facilities with a high probability of being granted a successful decision on their asylum applications. Further, this study focused exclusively on females, and the findings cannot be generalized to males — who represent the majority of refugees in Germany — as gender differences in mental disorders have been documented both in the general population and specifically among asylum seekers and refugees in prior research (84). The generalizability of the findings into groups living under other conditions, e.g. with less certainty in regard to their future, is therefore also limited.

The participants of our study had to fill out questionnaires. Participants from these countries who did not speak one of the target languages (e.g. Pashto or Kurdish speakers) were excluded based on the language inclusion criteria. Although questionnaires were translated and back-translated, there are still language differences due to various dialects or language comprehension. Illiterate women completed the questionnaires with the help of interpreters. Even though they were trained, the face-to-face situation is often intense, which might have influenced the interpreters, potentially affecting their objectivity and ability to maintain scientific detachment. Furthermore, the instruments used in our study are developed by western standards. There might be other culture-bound symptoms which can be seen as somatization symptoms or symptoms of distress that were not considered in our study. Therefore, instruments that are validated for different ethnic, cultural and language backgrounds are needed.

The countries of origin of the study participants are predominantly Islamic (85, 86). Therefore, the results cannot be readily generalized to female refugees from non-Muslim countries, such as Ukraine, where Christianity is the predominant religion (87). Our approach assessed questionnaire-based symptoms, appropriate to an overall epidemiological picture, but not for precise individual diagnosis. For a deeper understanding, an additional diagnosis of PTSD could provide more information, as participants reported traumatic events, which could also result in PTSD. It is also important to know whether the traumatic experiences occurred recently or much earlier in the participants’ lives. However, even after arriving in the host country, refugees face numerous challenges that affect their mental health, resilience, and quality of life such as the asylum process, language barriers, forced family separation, financial hardship, housing difficulties, cultural dissonance, discrimination, and everyday racism (88–90). These post-migration stressors should be systematically considered in future research.

Future research should focus on predictor variables investigating the relationship between trauma type and somatization symptoms and psychological distress. There is especially a need for more longitudinal high-quality research and further evaluation across diverse settings and populations. This study especially sheds more light on the psychological well-being of female refugees and highlights the need to include the gender perspective in diagnosis and intervention strategies.

## 5 Conclusion

Our research sheds light on the internal process through which traumatic events affect quality of life in female refugees. Inparticular, the impact of traumatic experiences on somatization and psychological distress leading to a reduced quality of life and worse mental health.

The finding is of high clinical relevance and highlights mechanisms of impaired quality of life in refugees. Therefore, when asylum seekers and refugees disclose trauma-related experiences, clinical assessments should be expanded to include integrated culturally sensitive tools screening for both somatic and psychological distress. Clinicians must be aware that the experience and expression of trauma can vary significantly across cultural groups and gender. They should also be particularly attentive when physical or somatic symptoms are reported, as these may indicate underlying traumatic experiences. Further studies should investigate more details of the interplay of somatization and distress. In particular, they should include more groups of refugees, as we only examined a selected number of countries of origin, and incorporate futher assessment of traumatic experience, for example including partner or domestic violence.

## Data Availability

The raw data supporting the conclusions of this article will be made available by the authors, without undue reservation.

## References

[1] United Nations High Commissioner for Refugees . Global trends: Forced displacement in 2024 (2025). Available online at: https://www.unhcr.org/sites/default/files/2025-06/global-trends-report-2024.pdf (Accessed October 10, 2025).

[2] Statistisches Bundesamt . 4 % mehr Schutzsuchende im Jahr 2024 (2025). Available online at: https://www.destatis.de/DE/Presse/Pressemitteilungen/2025/06/PD25_234_125.html (Accessed September 20, 2025).

[3] Kraus EK Sauer L Wenzel L . Together or apart? Spousal migration and reunification practices of recent refugees to Germany. J Family Res. (2019) 31:303–32. doi: 10.3224/zff.v31i3.04

[4] Damir-Geilsdorf S Sabra M . Disrupted families : the gendered impacts of family reunification policies on Syrian refugees in Germany. New York: UN Women (2018).

[5] Buhmann CB . Traumatized refugees: morbidity, treatment and predictors of outcome. Danish Med J. (2014) 61(8):B4871. Available online at: https://content.ugeskriftet.dk/sites/default/files/scientific_article_files/2018-11/b4871.pdf., PMID: 25162447

[6] Roupetz S Garbern S Michael S Bergquist H Glaesmer H Bartels SA . Continuum of sexual and gender-based violence risks among Syrian refugee women and girls in Lebanon. BMC Womens Health. (2020) 20:176. doi: 10.1186/s12905-020-01009-2, PMID: 32795272 PMC7427881

[7] Mundy SS Foss SLW Poulsen S Hjorthøj C Carlsson J . Sex differences in trauma exposure and symptomatology in trauma-affected refugees. Psychiatry Res. (2020) 293:113445. doi: 10.1016/j.psychres.2020.113445, PMID: 32977049

[8] Hassan G Ventevogel P Jefee-Bahloul H Barkil-Oteo A Kirmayer LJ . Mental health and psychosocial wellbeing of Syrians affected by armed conflict. Epidemiol Psychiatr Sci. (2016) 25:129–41. doi: 10.1017/s2045796016000044, PMID: 26829998 PMC6998596

[9] Schweitzer RD Vromans L Brough M Asic-Kobe M Correa-Velez I Murray K . Recently resettled refugee women-at-risk in Australia evidence high levels of psychiatric symptoms: individual, trauma and post-migration factors predict outcomes. BMC Med. (2018) 16:149. doi: 10.1186/s12916-018-1143-2, PMID: 30223855 PMC6142688

[10] Nesterko Y Jäckle D Friedrich M Holzapfel L Glaesmer H . Factors predicting symptoms of somatization, depression, anxiety, post-traumatic stress disorder, self-rated mental and physical health among recently arrived refugees in Germany. Confl Health. (2020) 14:44. doi: 10.1186/s13031-020-00291-z, PMID: 32670398 PMC7346670

[11] Çetrez >Ö.A DeMarinis V Sundvall M Fernandez-Gonzalez M Borisova L Titelman D . A public mental health study among Iraqi refugees in Sweden: social determinants, resilience, gender, and cultural context. Front Sociology. (2021) 6:551105. doi: 10.3389/fsoc.2021.551105, PMID: 33981759 PMC8109031

[12] Charlson F van Ommeren M Flaxman A Cornett J Whiteford H Saxena S . New WHO prevalence estimates of mental disorders in conflict settings: a systematic review and meta-analysis. Lancet. (2019) 394:240–8. doi: 10.1016/s0140-6736(19)30934-1, PMID: 31200992 PMC6657025

[13] Steel Z Chey T Silove D Marnane C Bryant RA van Ommeren M . Association of torture and other potentially traumatic events with mental health outcomes among populations exposed to mass conflict and displacement. JAMA. (2009) 302:537. doi: 10.1001/jama.2009.1132, PMID: 19654388

[14] Bogic M Njoku A Priebe S . Long-term mental health of war-refugees: a systematic literature review. BMC Int Health Hum Rights. (2015) 15:29. doi: 10.1186/s12914-015-0064-9, PMID: 26510473 PMC4624599

[15] Kessler RC Aguilar-Gaxiola S Alonso J Benjet C Bromet EJ Cardoso G . Trauma and PTSD in the WHO world mental health surveys. Eur J Psychotraumatol. (2017) 8:1353383. doi: 10.1080/20008198.2017.1353383, PMID: 29075426 PMC5632781

[16] Coventry PA Meader N Melton H Temple M Dale H Wright K . Psychological and pharmacological interventions for posttraumatic stress disorder and comorbid mental health problems following complex traumatic events: Systematic review and component network meta-analysis. PloS Med. (2020) 17:e1003262. doi: 10.1371/journal.pmed.1003262, PMID: 32813696 PMC7446790

[17] Schlaudt VA Bosson R Williams MT German B Hooper LM Frazier V . Traumatic experiences and mental health risk for refugees. Int J Environ Res Public Health. (2020) 17:1943. doi: 10.3390/ijerph17061943, PMID: 32188119 PMC7143439

[18] Starck A Gutermann J Schouler-Ocak M Jesuthasan J Bongard S Stangier U . The relationship of acculturation, traumatic events and depression in female refugees. Front Psychol. (2020) 11:906. doi: 10.3389/fpsyg.2020.00906, PMID: 32528358 PMC7247808

[19] Oppedal B Özer S Şirin SR . Traumatic events, social support and depression: Syrian refugee children in Turkish camps. Vulnerable Child Youth Stud. (2018) 13:46–59. doi: 10.1080/17450128.2017.1372653

[20] Barlattani T Renzi G D’Amelio C Pacitti F . Acute depressive reaction as a consequence of war trauma exposure: a case report of a Ukrainian refugee. Rivista di Psichiatria. (2023) 58:237–40. doi: 10.1708/4113.41073, PMID: 37807869

[21] Jongedijk RA Eising DD van der Aa N Kleber RJ Boelen PA . Severity profiles of posttraumatic stress, depression, anxiety, and somatization symptoms in treatment seeking traumatized refugees. J Affect Disord. (2020) 266:71–81. doi: 10.1016/j.jad.2020.01.077, PMID: 32056948

[22] Schneider A Pfeiffer A Conrad D Elbert T Kolassa I-T Wilker S . Does cumulative exposure to traumatic stressors predict treatment outcome of community-implemented exposure-based therapy for PTSD? Eur J Psychotraumatol. (2020) 11:1789323. doi: 10.1080/20008198.2020.1789323, PMID: 33062203 PMC7534285

[23] Vallejo-Martín M Sánchez Sancha A Canto JM . Refugee women with a history of trauma: gender vulnerability in relation to post-traumatic stress disorder. Int J Environ Res Public Health. (2021) 18:4806. doi: 10.3390/ijerph18094806, PMID: 33946312 PMC8125581

[24] Blackmore R Boyle JA Fazel M Ranasinha S Gray KM Fitzgerald G . The prevalence of mental illness in refugees and asylum seekers: A systematic review and meta-analysis. PloS Med. (2020) 17:e1003337. doi: 10.1371/journal.pmed.1003337, PMID: 32956381 PMC7505461

[25] Leiler A Bjärtå A Ekdahl J Wasteson E . Mental health and quality of life among asylum seekers and refugees living in refugee housing facilities in Sweden. Soc Psychiatry Psychiatr Epidemiol. (2018) 54:543–51. doi: 10.1007/s00127-018-1651-6, PMID: 30580381

[26] Rohlof HG Knipscheer JW Kleber RJ . Somatization in refugees: a review. Soc Psychiatry Psychiatr Epidemiol. (2014) 49:1793–804. doi: 10.1007/s00127-014-0877-1, PMID: 24816685

[27] Barakovic D Avdibegovic E Sinanovic O . Depression, anxiety and somatization in women with war missing family members. Materia Socio Med. (2013) 25:199–202. doi: 10.5455/msm.2013.25.199-202, PMID: 24167436 PMC3804435

[28] Morina N Schnyder U Klaghofer R Müller J Martin-Soelch C . Trauma exposure and the mediating role of posttraumatic stress on somatic symptoms in civilian war victims. BMC Psychiatry. (2018) 18:92. doi: 10.1186/s12888-018-1680-4, PMID: 29631551 PMC5891991

[29] Borho A Morawa E Schmitt GM Erim Y . Somatic distress among Syrian refugees with residence permission in Germany: analysis of a cross-sectional register-based study. BMC Public Health. (2021) 21:896. doi: 10.1186/s12889-021-10731-x, PMID: 33975567 PMC8114491

[30] Zbidat A Georgiadou E Borho A Erim Y Morawa E . The perceptions of trauma, complaints, somatization, and coping strategies among Syrian refugees in Germany—A qualitative study of an at-risk population. Int J Environ Res Public Health. (2020) 17:693. doi: 10.3390/ijerph17030693, PMID: 31973104 PMC7037213

[31] Rometsch C Denkinger JK Engelhardt M Windthorst P Graf J Gibbons N . Pain, somatic complaints, and subjective concepts of illness in traumatized female refugees who experienced extreme violence by the “Islamic State (IS). J Psychosom Res. (2020) 130:109931. doi: 10.1016/j.jpsychores.2020.109931, PMID: 31981895

[32] Löwe B Spitzer RL Williams JBW Mussell M Schellberg D Kroenke K . Depression, anxiety and somatization in primary care: syndrome overlap and functional impairment. Gen Hosp Psychiatry. (2008) 30:191–9. doi: 10.1016/j.genhosppsych.2008.01.001, PMID: 18433651

[33] Loeb TB Joseph NT Wyatt GE Zhang M Chin D Thames A . Predictors of somatic symptom severity: The role of cumulative history of trauma and adversity in a diverse community sample. Psychol Trauma. (2018) 10:491–8. doi: 10.1037/tra0000334, PMID: 29154595 PMC6021222

[34] Nissen A Hynek KA Scales D Hilden PK Straiton M . Chronic pain, mental health and functional impairment in adult refugees from Syria resettled in Norway: a cross-sectional study. BMC Psychiatry. (2022) 22:571. doi: 10.1186/s12888-022-04200-x, PMID: 36002823 PMC9404590

[35] Elklit A Christiansen DM . Predictive factors for somatization in a trauma sample. Clin Pract Epidemiol Ment Health. (2009) 5:1. doi: 10.1186/1745-0179-5-1, PMID: 19126224 PMC2632627

[36] Hiar S Thomas CL Hinton DE Salles J Goutaudier N Olliac B . Somatic symptoms mediate the relationship between trauma during the arab spring and quality of life among Tunisians. J Nervous Ment Dis. (2016) 204:153–5. doi: 10.1097/nmd.0000000000000446, PMID: 26825265

[37] Gentsch A Kuehn E . Clinical manifestations of body memories: the impact of past bodily experiences on mental health. Brain Sci. (2022) 12(5):594. doi: 10.3390/brainsci12050594, PMID: 35624981 PMC9138975

[38] Opaas M Wentzel-Larsen T Varvin S . The 10-year course of mental health, quality of life, and exile life functioning in traumatized refugees from treatment start. PloS One. (2020) 15:e0244730. doi: 10.1371/journal.pone.0244730, PMID: 33382807 PMC7775068

[39] Gagliardi J Brettschneider C König H-H . Health-related quality of life of refugees: a systematic review of studies using the WHOQOL-Bref instrument in general and clinical refugee populations in the community setting. Confl Health. (2021) 15:44. doi: 10.1186/s13031-021-00378-1, PMID: 34078413 PMC8173726

[40] Kounou KB Brodard F Gnassingbe A Dogbe Foli AA Sager JC Schmitt L . Posttraumatic stress, somatization, and quality of life among ivorian refugees. J Trauma Stress. (2017) 30:682–9. doi: 10.1002/jts.22244, PMID: 29194763

[41] Renner W Salem I . Post-traumatic stress in asylum seekers and refugees from chechnya, Afghanistan, and west africa: gender differences in symptomatology and coping. Int J Soc Psychiatry. (2009) 55:99–108. doi: 10.1177/0020764008092341, PMID: 19240200

[42] Taha PH Sijbrandij M . Gender differences in traumatic experiences, PTSD, and relevant symptoms among the Iraqi internally displaced persons. Int J Environ Res Public Health. (2021) 18:9779. doi: 10.3390/ijerph18189779, PMID: 34574702 PMC8471220

[43] Jesuthasan J Sönmez E Abels I Kurmeyer C Gutermann J Kimbel R . Near-death experiences, attacks by family members, and absence of health care in their home countries affect the quality of life of refugee women in Germany: a multi-region, cross-sectional, gender-sensitive study. BMC Med. (2018) 16:15. doi: 10.1186/s12916-017-1003-5, PMID: 29391012 PMC5793395

[44] Mollica RF Wyshak G de Marneffe DK Khuon F Lavelle J . Indochinese versions of the Hopkins Symptom Checklist-25: a screening instrument for the psychiatric care of refugees. Am J Psychiatry. (1987) 144(4):497–500. doi: 10.1176/ajp.144.4.497, PMID: 3565621

[45] Petermann F Brähler E . HSCL-25 hopkins-symptom-checkliste-25 – deutsche version. 1st edn. Göttingen: Hogrefe Verlag (2013).

[46] Petermann F Schmidt S . HSCL-25 hopkins-symptom-checkliste 25 - deutsche version. In: Geue K Strauß B Brähler E , editors. Diagnostische verfahren in der psychotherapie. Hogrefe Verlag, Göttingen (2016). p. 253–7.

[47] Glaesmer H Braehler E Grande G Hinz A Petermann F Romppel M . The German Version of the Hopkins Symptoms Checklist-25 (HSCL-25) — Factorial structure, psychometric properties, and population-based norms. Compr Psychiatry. (2014) 55:396–403. doi: 10.1016/j.comppsych.2013.08.020, PMID: 24286991

[48] Franke GH . Die symptom-checkliste von derogatis (SCL-90-R) - deutsche version - manual. 2nd edn. Göttingen: Beltz Test (2002).

[49] Hessel A Schumacher J Geyer M Brähler E . Symptom-checkliste SCL-90-R. Diagnostica. (2001) 47:27–39. doi: 10.1026//0012-1924.47.1.27

[50] Mollica RF Caspi-Yavin Y Bollini P Truong T Tor S Lavelle J . The harvard trauma questionnaire. J Nerv Ment Dis. (1992) 180:111–6. doi: 10.1097/00005053-199202000-00008 1737972

[51] Rasmussen A Verkuilen J Ho E Fan Y . Posttraumatic stress disorder among refugees: Measurement invariance of Harvard Trauma Questionnaire scores across global regions and response patterns. Psychol Assess. (2015) 27:1160–70. doi: 10.1037/pas0000115, PMID: 25894706 PMC4615261

[52] Vindbjerg E Carlsson J Mortensen EL Makransky G Nielsen T . A Rasch-based validity study of the Harvard Trauma Questionnaire. J Affect Disord. (2020) 277:697–705. doi: 10.1016/j.jad.2020.08.071, PMID: 32911220

[53] Hollifield M Warner TD Lian N Krakow B Jenkins JH Kesler J . Measuring trauma and health status in refugees. JAMA. (2002) 288:611. doi: 10.1001/jama.288.5.611, PMID: 12150673

[54] Sigvardsdotter E Malm A Tinghög P Vaez M Saboonchi F . Refugee trauma measurement: a review of existing checklists. Public Health Rev. (2016) 37:10. doi: 10.1186/s40985-016-0024-5, PMID: 29450052 PMC5809946

[55] Mollica RF Harvard Program in Refugee Trauma . Measuring trauma, measuring torture: instructions and guidance on the utilization of the Harvard Program in Refugee Trauma’s versions of The Hopkins Symptom Checklist-25 (HSCL-25) & the Harvard Trauma Questionnaire (HTQ). Cambridge, MA: Harvard Program in Refugee Trauma (2004).

[56] Harvard Program in Refugee Trauma . Harvard trauma questionnaire (2025). Available online at: https://hprt-cambridge.org/screening/harvard-trauma-questionnaire/ (Accessed October 10, 2025).

[57] Foa EB Cashman L Jaycox L Perry K . The validation of a self-report measure of posttraumatic stress disorder: The Posttraumatic Diagnostic Scale. Psychol Assess. (1997) 9:445–51. doi: 10.1037/1040-3590.9.4.445

[58] Norris AE Aroian KJ . Assessing reliability and validity of the Arabic language version of the Post-traumatic Diagnostic Scale (PDS) symptom items. Psychiatry Res. (2008) 160:327–34. doi: 10.1016/j.psychres.2007.09.005, PMID: 18718671 PMC2683387

[59] Brähler E Mühlan H Albani C Schmidt S . Teststatistische Prüfung und Normierung der deutschen Versionen des EUROHIS-QOL Lebensqualität-Index und des WHO-5 Wohlbefindens-Index. Diagnostica. (2007) 53:83–96. doi: 10.1026/0012-1924.53.2.83

[60] Schmidt S Mühlan H Power M . The EUROHIS-QOL 8-item index: psychometric results of a cross-cultural field study. Eur J Public Health. (2005) 16:420–8. doi: 10.1093/eurpub/cki155, PMID: 16141303

[61] Rocha NS Power MJ Bushnell DM Fleck MP . The EUROHIS-QOL 8-item index: comparative psychometric properties to its parent WHOQOL-BREF. Value Health. (2012) 15:449–57. doi: 10.1016/j.jval.2011.11.035, PMID: 22583455

[62] von Lersner U Elbert T Neuner F . Mental health of refugees following state-sponsored repatriation from Germany. BMC Psychiatry. (2008) 8:88. doi: 10.1186/1471-244x-8-88, PMID: 19000300 PMC2596775

[63] Carlsson JM Mortensen EL Kastrup M . Predictors of mental health and quality of life in male tortured refugees. Nord J Psychiatry. (2006) 60:51–7. doi: 10.1080/08039480500504982, PMID: 16500800

[64] Georgiadou E Schmitt GM Erim Y . Does the separation from marital partners of Syrian refugees with a residence permit in Germany have an impact on their quality of life? J Psychosom Res. (2020) 130:109936. doi: 10.1016/j.jpsychores.2020.109936, PMID: 31972478

[65] Walther L Fuchs LM Schupp J von Scheve C . Living conditions and the mental health and well-being of refugees: evidence from a large-scale german survey. J Immigr Minor Health. (2020) 22:903–13. doi: 10.1007/s10903-019-00968-5, PMID: 31974927 PMC7441051

[66] Renner A Jäckle D Nagl M Hoffmann R Röhr S Jung F . Predictors of psychological distress in Syrian refugees with posttraumatic stress in Germany. PloS One. (2021) 16:e0254406. doi: 10.1371/journal.pone.0254406, PMID: 34347775 PMC8336813

[67] Hayes A . Introduction to mediation, moderation, and conditional process analysis: A regression-based approach. New York: The Guilford Press (2018).

[68] Field A . Discovering statistics using IBM SPSS statistics. 5th edn. London: SAGE Publications Ltd (2018).

[69] Preacher KJ Hayes AF . SPSS and SAS procedures for estimating indirect effects in simple mediation models. Behav Res Methods Instruments Comput. (2004) 36:717–31. doi: 10.3758/bf03206553, PMID: 15641418

[70] Preacher KJ Rucker DD Hayes AF . Addressing moderated mediation hypotheses: theory, methods, and prescriptions. Multivariate Behav Res. (2007) 42:185–227. doi: 10.1080/00273170701341316, PMID: 26821081

[71] Araya M Chotai J Komproe IH de Jong JTVM . Effect of trauma on quality of life as mediated by mental distress and moderated by coping and social support among postconflict displaced Ethiopians. Qual Life Res. (2007) 16:915–27. doi: 10.1007/s11136-007-9201-9, PMID: 17440829

[72] Dangmann CR Solberg Ø Steffenak AKM Høye S Andersen PN . Health-related quality of life in young Syrian refugees recently resettled in Norway. Scand J Public Health. (2020) 48:688–98. doi: 10.1177/1403494820929833, PMID: 32613905 PMC7604933

[73] Macintyre K . An overview of somatization in the refugee population (2021). University of Virginia: Department of Family Medicine. Available online at: https://med.virginia.edu/family-medicine/wp-content/uploads/sites/285/2021/07/MacIntyre-Kara-Refugee-Paper_Finalpdf.pdf (Accessed October 10, 2025).

[74] Moran JK Jesuthasan J Schalinski I Kurmeyer C Oertelt-Prigione S Abels I . Traumatic life events and association with depression, anxiety, and somatization symptoms in female refugees. JAMA Netw Open. (2023) 6:E2324511. doi: 10.1001/jamanetworkopen.2023.24511, PMID: 37471088 PMC10359962

[75] Hinton DE Bui E . Variability of PTSD and trauma-related disorders across cultures: A study of Cambodians. In: Maercker A Heim E Kirmayer LJ , editors. Cultural clinical psychology and PTSD. Hogrefe, Göttingen (2019). p. 23–39.

[76] Brodda Jansen G . Two patient cases illustrating the importance of addressing physical and mental trauma as a cause of pain in refugee women. Front Sociology. (2020) 5:12. doi: 10.3389/fsoc.2020.00012, PMID: 33869421 PMC8022814

[77] Williams AC de C Peña CR Rice ASC . Persistent pain in survivors of torture: A cohort study. J Pain Symptom Manage. (2010) 40:715–22. doi: 10.1016/j.jpainsymman.2010.02.018, PMID: 20678891

[78] Sambucini D Aceto P Begotaraj E Lai C . Efficacy of psychological interventions on depression anxiety and somatization in migrants: A meta-analysis. J Immigr Minor Health. (2020) 22:1320–46. doi: 10.1007/s10903-020-01055-w, PMID: 32712851 PMC7683473

[79] Turrini G Purgato M Cadorin C Bartucz M Cristofalo D Gastaldon C . Comparative efficacy and acceptability of psychosocial interventions for PTSD, depression, and anxiety in asylum seekers, refugees, and other migrant populations: a systematic review and network meta-analysis of randomised controlled studies. Lancet Regional Health - Europe. (2024) 48:101152. doi: 10.1016/j.lanepe.2024.101152, PMID: 39687671 PMC11647468

[80] Mollica RF McInnes K Pool C Tor S . Dose-effect relationships of trauma to symptoms of depression and post-traumatic stress disorder among Cambodian survivors of mass violence. Br J Psychiatry. (1998) 173:482–8. doi: 10.1192/bjp.173.6.482, PMID: 9926076

[81] Atrooz F Khabour OF Almomani F Aljararwah S Alfurjani BH Salim S . Education and socioeconomic status as predictors of refugee mental health: insights from a study of Jordan-based Syrian refugee sample. Front Public Health. (2024) 12:1432205. doi: 10.3389/fpubh.2024.1432205, PMID: 39444980 PMC11496067

[82] Nikolov P Salarpour L Titus D . Skill downgrading among refugees and economic immigrants in Germany. arXiv. (2021) [Preprint]. arXiv:2111.00319. doi: 10.48550/arXiv.2111.00319

[83] Waisman G Larsen B . Income, amenities and negative attitudes. IZA J Migration. (2016) 5. doi: 10.1186/s40176-016-0056-0

[84] Hajak VL Sardana S Verdeli H Grimm S . A systematic review of factors affecting mental health and well-being of asylum seekers and refugees in Germany. Front Psychiatry. (2021) 12:643704. doi: 10.3389/fpsyt.2021.643704, PMID: 33815176 PMC8012840

[85] National Statistics Office (NSO) Fafo AIS . Eritrea population and health survey 2010. Asmara: National Statistics Office and Fafo Institute for Applied International Studies (2013).

[86] CIA World Factbook . The world factbook: countries (2023). Washington, DC: Central Intelligence Agency. Available online at: https://www.cia.gov/the-world-factbook/about/archives/2023/countries/ (Accessed September 22, 2025).

[87] EPFL Graph Search . Religion in Ukraine (2025). Available online at: https://graphsearch.epfl.ch/en/concept/9673688 (Accessed September 22, 2025).

[88] Laufer A Böttche M Walg M Khatib A Maoz-Dotan C Hassan H . Salutogenic and pathogenic factors among young adult refugees in Germany: An exploratory study. J Refugee Stud. (2022) 35:968–87. doi: 10.1093/jrs/feab110

[89] Borho A Morawa E Schug C Erim Y . Perceived post-migration discrimination: the perspective of adolescents with migration background. Eur Child Adolesc Psychiatry. (2023) 32:2427–38. doi: 10.1007/s00787-022-02084-6, PMID: 36127567 PMC10682162

[90] Walg M Khatib A Laufer A Böttche M Maoz-Dotan C Hassan H . Post-migration stress, quality of life, and mental health among accompanied and unaccompanied young refugees in Germany: How do adolescents feel after fleeing? Stress Health. (2024) 40(4):e3378. doi: 10.1002/smi.3378, PMID: 38279696

